# Pre-procedural predictors for multiple clips in percutaneous edge-to-edge mitral valve repair

**DOI:** 10.1186/s43044-021-00191-4

**Published:** 2021-09-14

**Authors:** Mohammad El Garhy, Bernward Lauer, Björn Göbel, Lisa C. Costello-Boerrigter, Carsten Salomon, Harald Lapp, Marc-Alexander Ohlow

**Affiliations:** 1 Department of cardiology, Central Clinic Bad Berka, Robert-Koch Allee 9, 99437 Bad Berka, Germany; 2 Department of Cardiology, Minia University, Minia, Egypt; 3 Department of Cardiology, Clinic of Internal Medicine I, Universitätsherzzentrum Thüringen, Friedrich Schiller University Jena, Jena, Germany

**Keywords:** MitraClip, Regurgitation, Mitral

## Abstract

**Background:**

Percutaneous mitral valve (MV) clipping for mitral regurgitation (MR) revolutionized MV repair; however, valve anatomies and pathologies vary. Often multiple clips are required, and predicting this pre-procedurally would be useful. We evaluated pre-procedural predictors for multiple clips.

**Results:**

We retrospectively analyzed 127 severe MR patients treated by mitral clipping between January 2011 and August 2018. Patients were grouped according to the use of a single (group I) or multiple clips (group II) and pre-procedure echocardiographs compared. No demographic differences existed except group II had more males (68.1%) than group I (48.3%). Mean left atrial diameter was larger in group II, 51 ± 9 mm, than group I, 48 ± 5 mm, *p* = 0.026. Mean mitral annular diameter differed: 34 ± 4mm (group II) versus 33 ± 3 mm (group I), *p* = 0.017. The vena contracta was broader in group II than group I (6.6 ± 1 mm vs. 6 ± 0.9 mm, *p* = 0.001). Severe mitral annular calcification occurred more in group I (36.2%) than group II (10.1%), *p* = 0.0001. On multivariate analysis, vena contracta width correlated positively with multiple clips (*B* 0.125, *p* = 0.013), but severe annular calcification correlated inversely (*B* − 0.35, *p* = 0.002).

**Conclusions:**

Vena contracta width and severe annular calcification are factors to consider when planning MV clipping.

## Background

It is widely recognized that severe, functional mitral regurgitation (MR) is associated with considerable morbidity and high rates of mortality. Mitral valve (MV) repair via transcatheter mitral clipping is an attractive therapeutic approach to secondary MR in inoperable and high-risk patients. However, recent randomized clinical trials have yielded contradictory results in such patients [1, 2].

There are two common techniques for clip implantation: the central clip technique and the zipping technique. The central clip technique uses one clip central to the regurgitant jet, and the zipping technique uses multiple clips sequentially from medial to lateral [3]. The first technique is more common and is also the standard technique used at our institution. With this approach, it would be helpful to identify those patients who would require multiple clips in advance. Alegria-Bareiro et al. reported that a vena contracta > 7.5 mm and the presence of more than one broad jet are predictors for the use of multiple mitral clip devices [4]. This study also found that the presence of a restricted posterior mitral valve leaflet (PML) correlated inversely with the use of more than one clip [4]. Unfortunately, this study was small and included only 19 patients with secondary MR. Using a large database that our institution has maintained, we aimed to evaluate possible predictors for the need of multiple clips when treating severe secondary MR by the central clip technique.

## Methods

### Patient characteristics

This retrospective study was conducted in accordance with the principles of the Declaration of Helsinki and our institutional requirements. All patients were treated by percutaneous edge-to-edge repair using mitral clip devices for severe MR between January 2011 and August 2018 at our institution, which is a tertiary care hospital with a busy cardiovascular referral center. Patients were divided into two groups: Group I included patients who were treated with a single clip and group II included patients treated with more than one clip. We did not adhere to EVEREST exclusion criteria [5] and included mostly inoperable patients. We offered this as a palliative therapy to improve quality of life. In difficult cases, we used intra-aortic balloon pumps, rapid pacing, and ventilator maneuvers. We only excluded patients with a mean gradient > 4 mm Hg over the MV, active endocarditis, or reduced life expectancy (less than 1 year).

### Pre-procedure assessment

All patients underwent preoperative left and right heart catheterization and transthoracic (TTE) and transesophageal (TEE) echocardiography before the intervention to assess MV morphology, the severity of the MR, and the anatomical suitability for edge-to-edge repair with a mitral clip device such as MitraClip® (Abbott Vascular, Santa Clara, California, USA).

Resting echocardiographic images were reviewed to obtain left atrial and left and right ventricular dimensions and functions. Mitral annular diameter was measured in end-systole in parasternal and apical long-axis views. The assessment of MR was largely dependent upon the vena contracta (severe MR ≥ 7 mm and moderate MR > 4 mm and < 7 mm). However, factors such as left ventricular function and dimensions, pulmonary artery pressure, and patient's symptoms were also considered. Stress echocardiography was performed in some cases when the vena contracta was < 7 mm to define the significance of the MR better.

Clinical assessment included recording symptoms, New York Heart Association (NYHA) Functional Class, and the risk of conventional surgical repair.

### Percutaneous intervention and follow-up

The procedure was performed by a single experienced operator under general anesthesia in a hybrid operating room. After obtaining vascular access via the right femoral vein, the transseptal puncture was performed with TEE, and fluoroscopic guidance and heparin anticoagulation started. The edge-to-edge repair was completed under 3-D TEE guidance. Hemodynamics were monitored throughout the procedure via the radial artery and via a central venous catheter. Left atrial pressure was recorded both pre- and post-procedure. All procedures were performed according to the central clip concept [Alg REF?]. Additional clip implantation was attempted only if residual MR was more than grade 2 and the mean MV gradient < 5 mmHg. The patients were extubated in the hybrid operating room and monitored for at least 24 h in the intermediate care unit. Patients were then transferred to the cardiology ward and discharged on the fifth postoperative day. TTE was performed before discharge. All patients had a clinical follow-up, including TTE and TEE, at 3 months post-procedure.

### Statistical analysis

Continuous variables were expressed as means and standard deviations and categorical data as percentages. To identify differences between both groups, unpaired Student’s *t*-test was used for continuous variables and Fisher’s exact test for categorical variables. Variables that differed significantly between groups were entered into a multivariate binary logistic regression analysis. ROC curves were plotted for the parameters identified in the multivariate model as independent determinants for the utilization of two clips. All analyses were done using SPSS statistical software (IBM Corp. Released in 2013. IBM SPSS Statistics for Windows, Version 22.0. Armonk, NY: IBM Corp).

## Results

### Patient characteristics

A total of 127 patients were retrospectively reviewed. Fifty-eight patients (46%) were treated with a single clip (group I), and 69 patients (54%) required more than one clip (group II).

Male gender was more prevalent among patients requiring more than one clip (68% vs. 48%, *p* = 0.018). Otherwise, there were statistically no significant differences between the two groups regarding other baseline characteristics (Table 1). This study included very high-risk patients (age 77 ± 8 years, logEuroScore 19 ± 13, and STS 8.5 ± 2.5) who suffered from advanced heart failure (NYHA III-IV 91%) and were considered unsuitable candidates for surgery. Most of those patients had severely depressed left ventricular systolic function with secondary MR (82%). Only a minority of patients had primary MR, and they were equally distributed between groups, with 10 (17.2%) primary MR cases in group I and 13 (18.8%) in group II.

**Table 1:** Baseline preoperative characteristics

	Total	Group I	Group II	*p*-value
		*n* = 58	*n* = 69	
Age, years ± SD	77 ± 8	78 ± 7	77 ± 8	0.3
Male, *n* (%)	75 (59%)	28 (48%)	47 (68%)	0.01
NYHA III–IV, *n* (%)	117 (91%)	56 (97%)	61 (89%)	0.4
Log EuroScore, mean ± SD	19 ± 13	16 ± 9	21 ± 15	0.09

*NYHA* New York heart association, *N* number, *SD* standard deviation

### Predictors of need for multiple clips

**Fig. 1:**
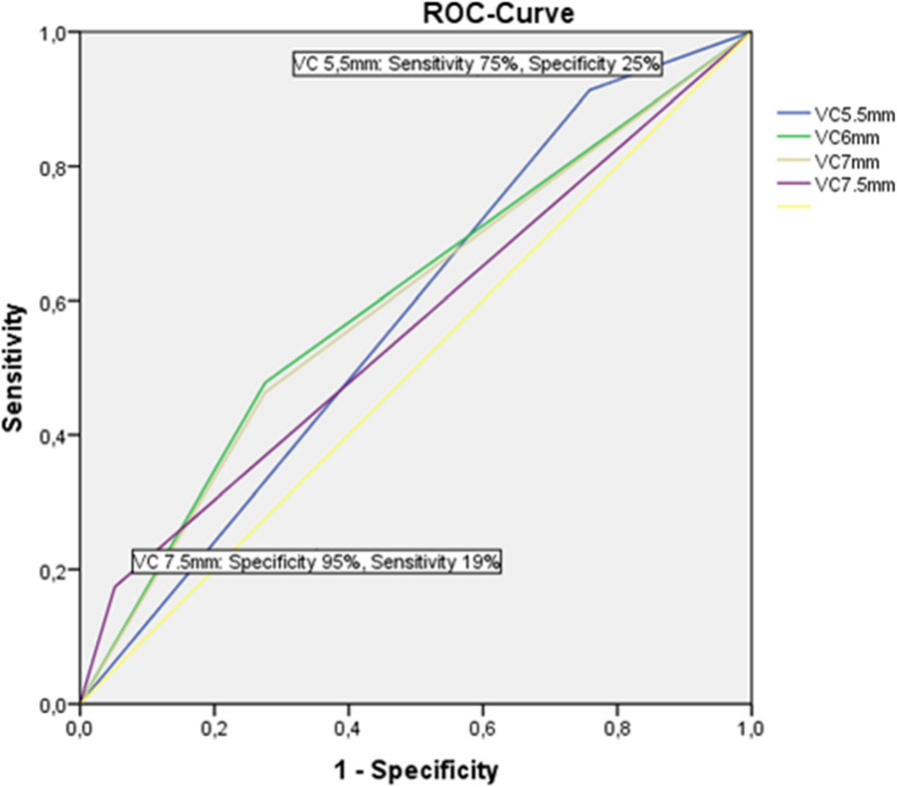
ROC curve shows the sensitivity and specificity of the different cutoff levels of VC to predict the use of multiple clips

Pre-intervention echocardiographic data are in Table 2. The mean MV orifice area was 3.9 ± 1.2 cm^2^ (group I) versus 3.8 ± 1.1 cm^2^ (group II), *p* = 0.9. Left atrial diameter and mitral annulus dilatation were larger in patients requiring > 1 clip. Mean left atrial diameter was 51 ± 9mm (group II) versus 48 ± 5mm (group I), *p* = 0.026, and mean mitral annulus diameter was 34 ± 4 mm (group II) versus 33 ± 3 mm (group I) *p* = 0.017. Also, the vena contracta was broader in group II compared to group I (6.6 ± 1 mm vs. 6 ± 0.9 mm, *p* = 0.001). Conversely, severe mitral annular calcification occurred significantly more often in those who required only one clip, being present in 36% of group I, but only in 10% of group II (*p* = 0.0001). The Carpentier classification of MR, according to leaflet motion (Table 1) was not a predictor of the use of multiple clips. In the multivariate analysis, vena contracta width correlated positively with the use of multiple clips (*B* 0.125, CI 95.0% 0.027–0.22, *p* = 0.013), and severe annular calcification correlated inversely with it (*B* − 0.35, CI 95.0% 0.13–0.57, *p* = 0.002). Using a ROC analysis, a vena contracta cutoff of 7.5 mm was specific but not sensitive (95% and 19%, respectively). Sensitivity and specificity for a vena contracta cutoff of 5.5 mm were 91% and 25%, respectively; for a cutoff of 6.5 mm, the values were 47% and 75%, respectively, Fig. 1.

**Table 2:** Echocardiographic preoperative characteristics

	Total	Group I	Group II	*p*-value
	*n* = 127	*n* = 58	*n* = 69	
Annular calcification, *n* (%)	28 (22%)	21 (36%)	7 (10%)	0.0001
Moderate to severe TR, *n* (%)	75 (59%)	33 (57%)	42 (61%)	0.8
Carpentier class:				
I, *n* (%)	28 (22%)	14 (24%)	14 (20%)	0.5
II, *n* (%)	9 (7%)	4 (7%)	5 (7%)	0.2
III, *n* (%)	90 (71%)	40 (69%)	50 (72%)	0.6
Multiple broad jets, *n* (%)	7 (6%)	2 (3%)	5 (7%)	0.6
Eccentric jet, *n* (%)	38 (30%)	16 (28%)	22 (32%)	0.8
PML < 10 mm, *n* (%)	21 (17%)	12 (21%)	9 (13%)	0.5
LVEDD, mm	58 ± 9	57 ± 10	59 ± 9	0.1
LA diameter, mm	49 ± 8	48 ± 5	51 ± 9	0.026
LVEF, %	38 ± 15	40 ± 15	37 ± 14	0.2
Annulus size, mm	34 ± 4	33 ± 3	34 ± 4	0.017
RVSP, mmHg	47 ± 14	45 ± 16	48 ± 18	0.3
Vena contracta, mm	6.3 ± 1	6.0 ± 0.9	6.6 ± 1	0.001

*TR* tricuspid regurgitation, *LVEDD* left ventricle end-diastolic diameter, *LVEF* left ventricle ejection fraction, *RVSP* right ventricle systolic pressure, *PML* posterior mitral leaflet, *LA* left atrial

**Fig. 2:**
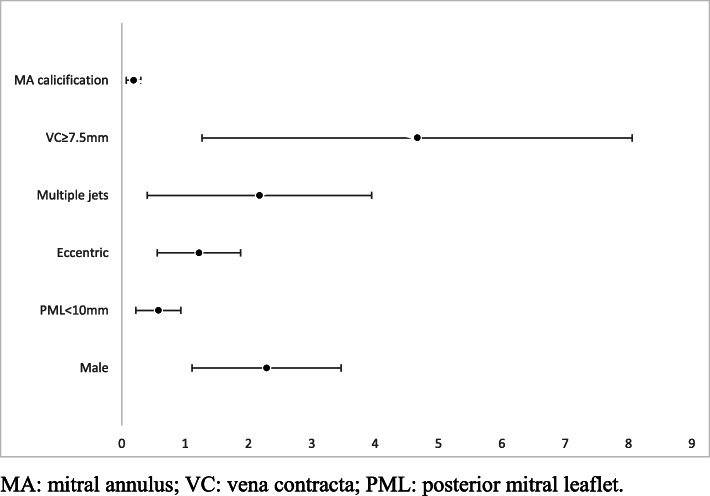
Odds ratios of the possible predictors of the use of multiple clips. MA, mitral annulus; VC, vena contracta; PML, posterior mitral leaflet

The odds ratios of the possible predictors are presented in Fig. 2.

### Follow-up

**Fig. 3:**
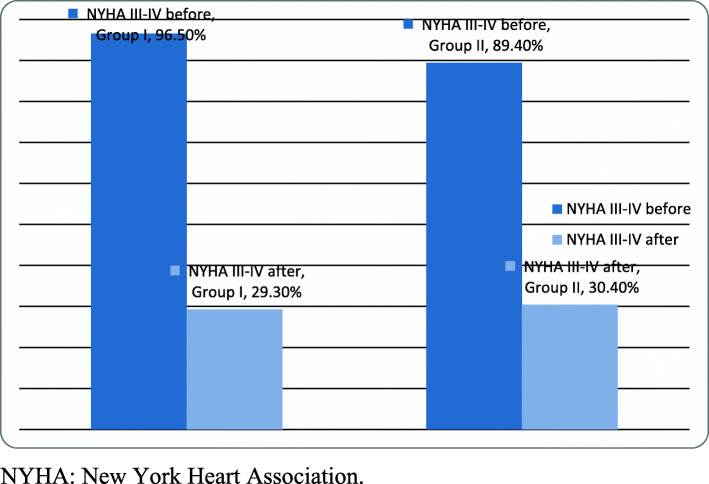
NYHA classification before and after mitral clip procedure. NYHA, New York Heart Association

At the 3-month follow-up, the incidence of all-cause mortality was 11%, of residual MR > grade 2 was 19.6%, of reintervention (clip or surgery) was 6.3%, and of procedural complication was 3.9%. MV clipping improved symptoms by at least one NYHA class in 51% of the study patients. There were no significant differences between the two groups regarding these endpoints (Table 3 and Fig. 3).

**Table 3:** Procedural and post-procedural characteristics

	Total	Group I	Group II	*p*-value
	*n* = 127	*n* = 58	*n* = 69	
Fluoroscopy time, min	9.8 ± 9.1	8.2 ± 8.3	11.1 ± 9.6	0.07
DLP, mGy*cm	2065 ± 4050	2406 ± 4725	1783 ± 3405	0.3
Mean PG after clip, mmHg	4.4 ± 1.79	4.3 ± 1.8	4.49 ± 1.79	0.6
MR > grade 2 after clip, *n* (%)	25 (19.6%)	12 (20.7%)	13 (18.8%)	0.6
NYHA III-IV after clip, *n* (%)	38 (30%)	17 (29.3%)	21 (30.4%)	0.5
Symptomatic improvement, *n* (%)	62 (50.6%)	30 (49.2%)	32 (52.2%)	0.6
Procedure related mortality, *n* (%)	5 (3.9%)	3 (5.2%)	2 (3.9%)	0.5
3-month mortality, *n* (%)	14 (11%)	8 (13.8%)	6 (8.7%)	0.2
ReIntervention, *n* (%)	8 (6.3%)	3 (5.2%)	5 (7.2%)	0.7

*DLP* dose length product, *MR* mitral regurgitation, *NYHA* New York Heart Association, *PG* pressure gradients

Before the mitral clipping, 75% of patients were treated with beta-blockers, 90% with ACEi/ARB/sacubitril + valsartan, 64% with aldosterone antagonists, and 78% with loop diuretics. During the period of follow up, the proportion of patients in both groups who received beta-blockers (75% group I vs. 74% group II), ACEi/ARB/sacubitril + valsartan (90% vs. 92%), and aldosterone antagonists (64% vs. 63%) and loop diuretics (78% vs. 70%) was unchanged when compared with baseline. The proportion of patients treated with loop diuretics was likewise unchanged; however, a decrease in daily diuretic dose was observed in 62 patients (48.8%); 29 patients in group I (51.7%); and 33 patients in group II (47.8%).

## Discussion

**Fig. 4:**
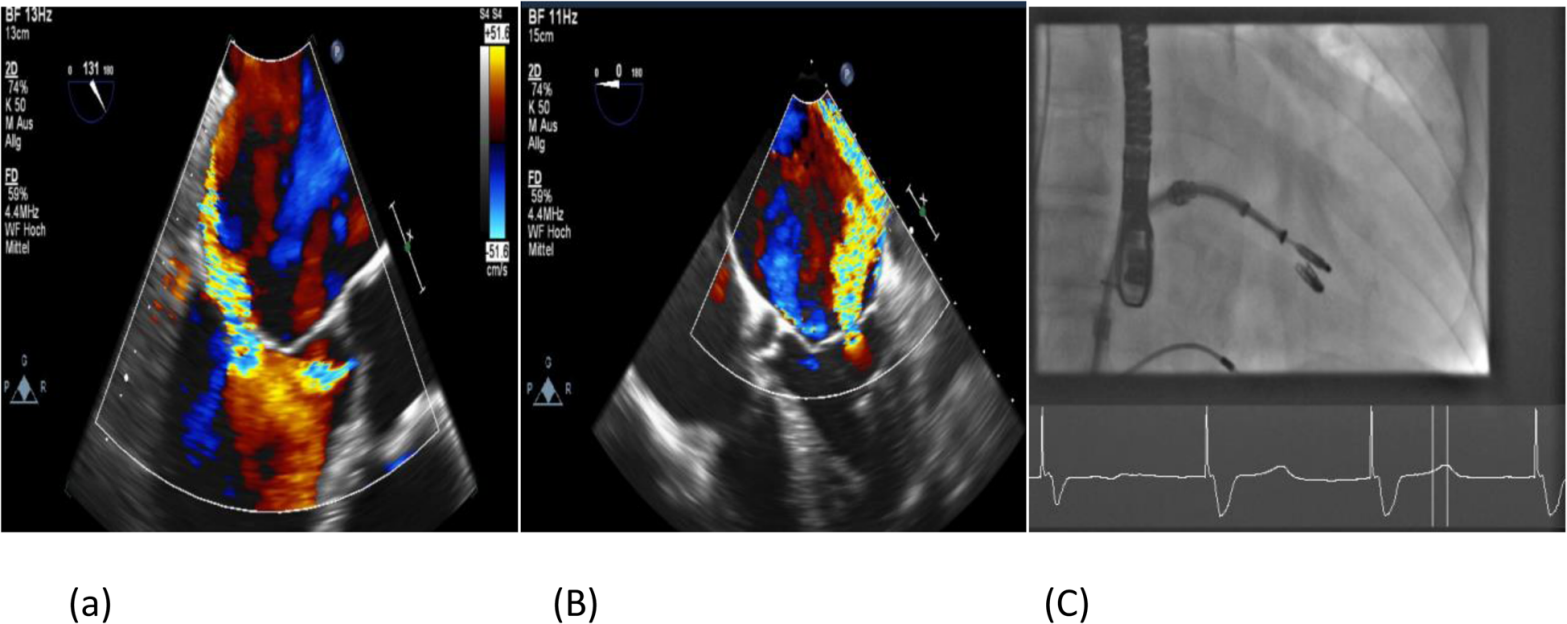
TEE showing MR with wide VC (9 mm) in 3-chamber (**a**) and 4-chamber (**b**) mid-esophageal view, which was treated with 2 clips (**c**)

**Fig. 5:**
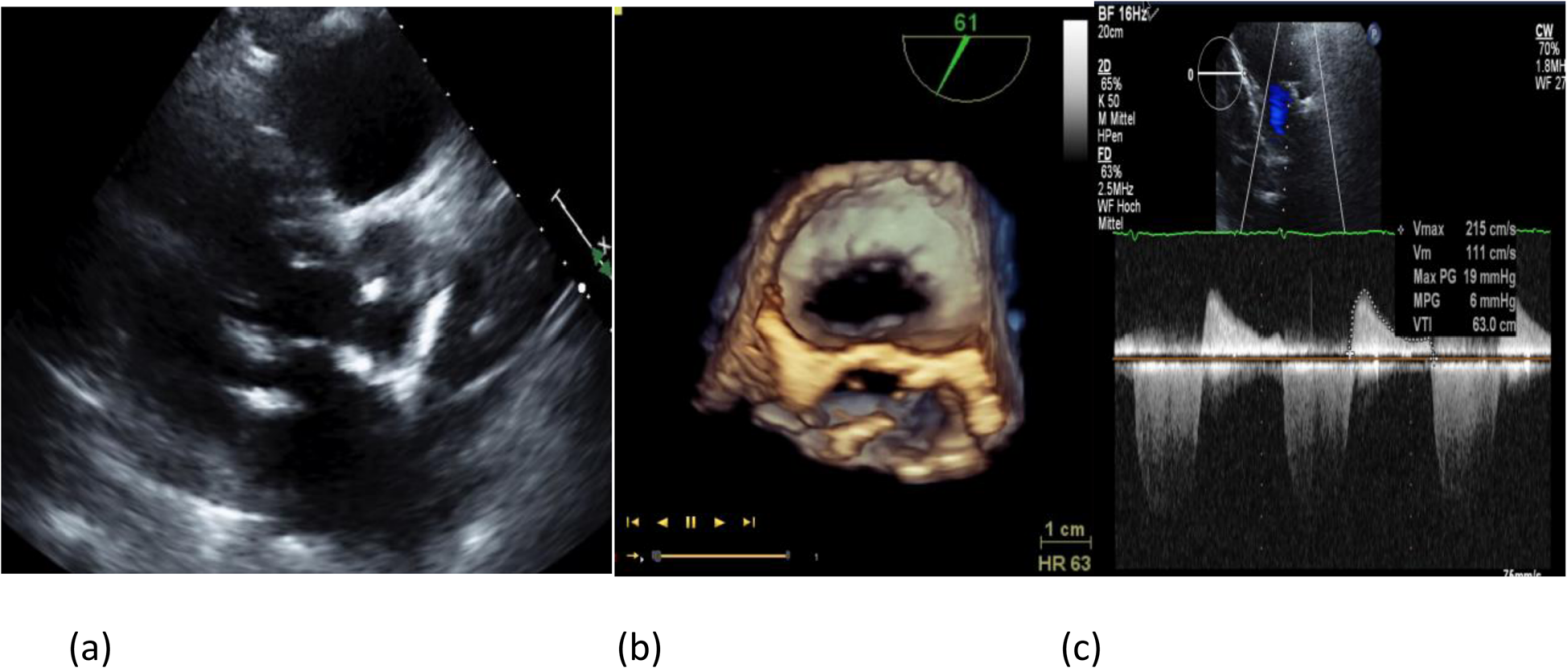
2D TTE showed annular calcification (**a**). Even though the mitral valve area was more than 3 cm^2^ in 3D (**b**), the mean gradient after the first clip was 6 mmHg with residual MR (**c**)

In this cohort of patients with severe MR who were unsuitable for surgery, we found that more than one mitral clip was needed in 69 patients (54%) and that vena contracta width was an independent predictor of the need for multiple clips (Fig. 4). A vena contracta width cutoff of 7.5 mm was highly specific (95%) but not sensitive (18%). However, the presence of mitral annular calcification correlated inversely with the use of multiple clips (see Fig. 5). Importantly, we found that the use of multiple clips was safe and not associated with a higher incidence of periprocedural complications. Comparing the one- and multiple-clip groups, there was no significant difference in terms of any of the composite endpoints (mortality, procedural complication, residual regurgitation, and reintervention).

Our study included mainly patients with secondary MR (82%). The minority of patients with primary MR were equally distributed between group I (17%) and group II (19%). Prior studies have reported on the use of MitraClip® in degenerative MR. However, two recent multicenter trials examined the use of MitraClip® in secondary MR and obtained contradictory results [1, 2].

In our study, the incidence of the need for more than one clip (54%) was consistent with what had previously been reported by Alergia et al. [4]. However, Giordano et al. observed a lower incidence of multiple clipping. Of note, in that small study, more of the patients were classified as Carpentier Class II (23.3%) [6]. In our larger study, only 7% of the patients were Carpentier Class II. Due to the more localized pathology in Carpentier Class II MR, such cases can often be targeted with one clip.

Our study corroborates the primary finding of Alegria-Bareiro et. al., specifically, that the width of the vena contracta predicts the necessity of using multiple clips [4].. The current guidelines differentiate between the quantification of primary and secondary MR [7]. Even the American and European guidelines are not consistent regarding when to diagnose severe, secondary MR [7, 8]. Most of our patients had secondary MR with an ERO between 0.2 and 0.4 cm^2^. The previously proposed 7.5-mm cutoff width [4] had an excellent specificity of 95% and a low sensitivity of 19%. Therefore, we recommend using the zipping technique in patients with secondary MR and vena contracta > 7.5 mm. We believe that a vena contracta width ≥ 7.5 mm is the best applicable cutoff in secondary MR because of the high specificity. Other researchers found no clear-cut features that could predict the need for multiple clips.

The new guidelines address the role of left atrial dilatation as a possible mechanism of secondary MR [7, 8]. In our study, the mean left atrial diameter and mean mitral annular diameter was significantly larger in those patients who required more than one clip. Conversely, neither the left ventricular diameter nor left ventricular function differed significantly between groups. Other parameters, such as left ventricular volume, coaptation depth, and sphericity index, were not available for the retrospective study. However, these parameters could be relevant here. Severe mitral annular calcification, which is mostly associated with extensive valve pathology and restricted mobility of the leaflets, correlates inversely with the use of more than one clip.

Fourteen patients (11%) died during the follow-up period of 3 months. These deaths were primarily due to terminal heart failure, as most of our patients had severely depressed left ventricular function at baseline. In previous studies, 6-month mortality ranged from 10 to 13%, and the 1-year mortality rate was between 6 and 24% [1–3, 9]. The EVEREST II Trial [5], which reported the lowest mortality rate (6%), included younger patients with a better functional class (mean age 67 years in EVEREST II vs. 77 years in our study, and NYHA III in 50% in EVEREST II vs. 91% in our study). Other studies, which included sicker patients with more secondary MR, have mortality rates in keeping with our study [1, 2].

Although mitral clipping significantly reduced MR to ≤ grade 2 in 80% of the study patients, it improved heart failure symptoms significantly in only 51%. This discrepancy can easily be explained by the fact that secondary MR is not so much a valvular disease; instead, it is the result of left ventricular abnormalities (e.g., dilated or ischemic cardiomyopathies) and associated issues with mitral annular dilatation and/or papillary muscle displacement. Thus, secondary to remodeling and dilatation of the left ventricle, the mitral valve does not coapt properly. In patients with secondary MR, even after the MR is reduced, the impaired left ventricular function remains. So it is unsurprising that heart failure symptoms do not improve to the same degree as the MR itself. Furthermore, we included high-risk patients with multiple comorbidities (coronary artery disease 34%, renal impairment 29%, and chronic lung disease 26%). Given that we assessed the symptomatic improvement only with NYHA class, a modest improvement in dyspnea could be missed in these patients.

The rate of MR reduction in our study is less than reported previously in the COAPT trial [2], but, as alluded to above, we performed these mitral clipping procedures as last-line therapy after failure of optimal medical and device therapies. In the COAPT trial, MR improved in 46% of patients in the control group, which might be explained by further optimization of medical therapy after randomization. Moreover, 11.7% of the intervention group in the COAPT trial received resynchronization therapy or mechanical support after clipping.

## Limitations

The limitations of this study are its retrospective and observational design, the lack of some echocardiographic data, and the absence of long-term follow-up.

## Conclusion

Vena contracta width is an independent predictor of the use of multiple clips in patients with secondary mitral regurgitation. Cutoff level VC ≥ 7.5mm offered high specificity but low sensitivity. Severe annular calcification correlates inversely with the use of > 1 clip. The use of multiple clips is safe and was not associated with a higher incidence of periprocedural complications.

## Data Availability

All data are available on request at the Department of Cardiology, Zentralklinik Bad Berka, Germany.

## Abbreviations

NYHA: New York Heart Association
MR: Mitral regurgitation
TTE: Transthoracic echocardiography
TEE: Transesophageal echocardiography
VC: Vena contracta
